# Characterization of reading processes in Brazilian adolescents: development of criteria for identifying students at risk for dyslexia

**DOI:** 10.3389/fpsyg.2025.1477896

**Published:** 2025-07-09

**Authors:** Renata Pires Sena de Assumpção Victorio, Giseli Donadon Germano

**Affiliations:** ^1^School of Philosophy and Sciences, São Paulo State University (UNESP), São Paulo, Brazil; ^2^Learning Disabilities Investigation Laboratory, School of Philosophy and Sciences, São Paulo State University (UNESP), São Paulo, Brazil

**Keywords:** dyslexia, reading, assessment, monitoring, adolescents

## Abstract

**Introduction:**

Reading processes in Brazilian pre-adolescent and adolescent students are scarcely investigated, and it is essential to identify gaps and possible dyslexic students that have not yet been determined. Therefore, the objective of this study was to characterize the reading processes in adolescents and develop criteria to identify Brazilian students at risk for dyslexia in middle school.

**Methods:**

Two hundred six students participated in the study, divided into four groups: 55 students (6th grade), 54 students (7th grade), 46 students (8th grade), and 51 students (9th grade). Initially, they were assessed collectively in the Evaluation of the Reading Processes Test. Criteria were developed, and the students were classified in relation to their reading performance as no risk, low risk (LR) and high risk (HR) for dyslexia. Only students who presented low or high risk were assessed individually, that is, 84 students. These were evaluated separately and classified in relation to accuracy and reading speed, meeting the criteria for risk for dyslexia. Finally, they were classified into phonological, visual and undefined profiles.

**Results:**

The collective phase revealed 27.3% LR and 12.7% with HR in GI, 29.6% with LR and 25.9% with HR in 7th grade, 23.9% with LR and 13% with HR in 8th grade, and 23.5% with LR and 5.9% with HR in 9th grade. As for profiles, 34.6% met the criteria for phonological profile, 23.8% for visual profile, 2.4% for mixed profile, and 39.3% of students did not fit the established profile criteria.

**Conclusion:**

Assessments are necessary for this population, as evidenced by the indication of students at risk for dyslexia who have not yet been diagnosed. Finally, the study reinforces the idea of the heterogeneous nature of dyslexia in pre-adolescent and adolescent students.

## 1 Introduction

Learning to read is a complex act that involves cognitive-linguistic skills that improve throughout life and schooling. Understanding reading processes and their development in pre-adolescents and adolescents is essential so that possible gaps can be monitored and students with reading learning disorders can be identified and referred to the health network. It is also necessary for educational institutions to provide academic support to students with dyslexia, adapting their pedagogical practices.

School failure and reading difficulties are often correlated in the literature since less fluent students may have problems understanding and assimilating content from the most diverse curricular subjects, negatively impacting their academic career (Bigozzi et al., 2017; Corso and Salles, 2009; Komeno et al., 2015; Martins and Capellini, 2019). It is not new that Brazilian and international reading performance assessment programs have recorded lower reading performance in pre-adolescent and adolescent students in Brazil (INEP, 2021; OCDE, 2023).

Recently, the Programme for International Student Assessment (PISA) published data on the performance of 15-year-old students in mathematics, reading and science. The results indicated that Brazil occupied the 52nd position in the reading ranking, with 50% of Brazilian students performing below the basic level in reading, which means that they have difficulty, for example, identifying explicit information in a text, that is, considered as the minimum to exercise full citizenship. Among OECD member countries, this figure was 27%. Furthermore, Brazil has yet to reach the maximum level of reading proficiency, and these young people are at the lowest level of the assessment (INEP, 2021; OCDE, 2023).

There is still a limited number of Brazilian studies with middle school and high school students, emphasizing this problem. Moreover, there is an absence of reading parameters in Brazil for this population and a scarcity of screening and assessment instruments for reading learning disorders for pre-adolescent and adolescent students available to Brazilian professionals (Alves et al., 2021; Gentilini et al., 2020; Pires et al., 2023).

Considering the fundamental role of learning to read, it becomes necessary as soon as entering elementary school. To learn to read, the child must develop the perception that graphic symbols can represent speech and that graphic symbols represent the sounds of the language (Scliar-Cabral, 2003). This perception develops through constant exposure to the writing system and the efficient teaching of alphabetic principles (Scliar-Cabral, 2003; Oliveira et al., 2016).

Furthermore, the various brain regions responsible for processing the skills involved in the acquisition and development of reading must be functioning properly and integrated with the activation of specific brain regions during reading, such as the occipital lobe, regions left inferior parietal areas and left temporal lobe, in addition to frontal regions responsible for eye movements and subcortical structures (Corso and Salles, 2009). When starting the literacy process, the learner masters the linguistic system in its oral form, using it as an instrument for expressing and understanding meanings. Language develops naturally through social interaction and experiencing the environment, being the basis for the development of written language (Scliar-Cabral, 2003). Efficient and automated reading depends on decoding capacity and the constitution of the orthographic lexicon. Brazilian Portuguese is an alphabetic language, which uses the alphabet as the basis for its writing. However, in terms of transparency, the Brazilian Portuguese writing system is relatively transparent, as it presents cases in which the letter (grapheme)/phoneme correspondences are not direct (Scliar-Cabral, 2003). As so, for Brazilian Portuguese there are also irregularities in grapheme-phoneme conversion due to reading practices (Lyon et al., 2003; Pinheiro et al., 2008; Pinheiro and Rothe-Neves, 2001).

Thus, reading automaticity is established on four main pillars: speed, absence of effort, autonomy and absence of conscious attention. Therefore, reading fluently means reading correctly and expressively, with an adequate reading speed. It, hence, combines elements of accuracy, correct decoding of orthographic signs; prosody, reading with appropriate rhythm and intonation; and automaticity, fast and natural reading (Alves et al., 2021; Hudson et al., 2005).

However, we observed a paradox with the assumption of the Common National Curriculum Base (BNCC; Brazil, 2017) in Brazil, which assumes an enunciative-discursive perspective of language, with a central focus on the text as a work unit and the development of skills for the meaningful use of language in reading activities. The document guiding educational practices highlights that reading and the search for autonomy and fluency with texts must be achieved in the early years of elementary school, that is, from the 1st to the 5th grade. Thus, as a consequence of the work carried out in previous stages of schooling, adolescents and young people already know and use different genres, seeking to develop a critical sense (Brazil, 2017).

Brazilian studies indicate that, once the literacy phase is over, in which students learn to decode orthographic signs, their reading matures and becomes automatic, being able to provide attention and memory resources for higher levels of reading, such as textual comprehension (Martins and Capellini, 2019; da Silva and da Fonseca, 2021). Fernandes et al. (2015) also report that learning to read is continuous and can extend beyond 18 years of age. In stage 1, students learn letter-sound correspondence rules (1st and 2nd grade); in stage 2, they improve their reading accuracy and speed, developing automaticity (2nd and 3rd grade); in stage 3 (4th to 9th grade), they are faced with increasingly greater demands, in terms of quantity and complexity, of expository texts; in stage 4 (high school) they are faced with multiple interpretations of a given text, and begin a process of evaluating these materials based on their knowledge of the subject; in stage 5, the final stage (graduation onwards), they refine their critical awareness, dealing with multiple points of view, building and reconstructing knowledge.

However, in Brazil, even though the BNCC recommends that students must complete middle school by reading texts with autonomy, fluency and criticality, many Brazilian studies have highlighted reading gaps in this educational cycle (de Andrade et al., 2019; Alves et al., 2021; Pires et al., 2024). At each level of education, there will be different demands on cognitive skills for reading comprehension to be achieved. Therefore, students with reading gaps can be identified late in more advanced cycles of basic education. Moreover, among those with academic losses due to difficulties in reading comprehension and assimilation of syllabus content, we will be able to identify not only students who carry deficits in phonological skills from the initial years, such as lexical recognition but also those who have failed to mature in other cognitive skills necessary for reading comprehension, with the increase in linguistic demand in the final years (Chang and De Avila, 2014).

Therefore, understanding the reading processes for the adolescent population is also important, as students with reading learning disorders or dyslexia may not have been identified throughout elementary school. This Brazilian educational context is completely unfavorable for the identification of students with dyslexia since fluency is not monitored in the initial years of literacy, and reading failures are not seen as an indicator of dyslexia, as they are “common” manifestations in the classroom. However, we know that dyslexia can have serious implications in adulthood due to the persistent deficit of reading difficulties. Indeed, when neglected, dyslexia can lead to low levels of education and high rates of unskilled employment (Snowling et al., 2020).

The Diagnostic and Statistical Manual of Mental Disorders (DSM-V, APA, 2014) describes dyslexia as a specific learning disorder characterized by persistent difficulties in reading ability, such as inefficient letter/sound decoding., slow and imprecise reading, and impairments in reading comprehension. The manual also foresees academic skills below those expected for chronological age, with possible losses in school performance, even in the presence of adequate educational instruction.

In addition, dyslexia may be caused by a genetically imposed dysfunction of one or more specific brain modules that are critically important for the acquisition of reading ability (Pennington, 1991). According to studies, dyslexia can affect between 3 and 10% of school-age children, with a higher incidence in males. In Brazil, it can reach 5–15% of students. It is characterized by failures in accuracy and speed in oral reading, which may or may not compromise reading comprehension and spelling (Mantovani et al., 2021; Verwimp et al., 2023). A national study carried out on an elementary school population has already highlighted the heterogeneous nature of dyslexia in Brazilian schoolchildren, drawing attention to reading impairments, which may result not only from phonological failures but also from visual-attentional failures (Germano et al., 2014).

Among the indicators for dyslexia, the lack of monitoring of word reading and reading fluency measures makes it difficult to identify this population early (APA, 2014; Santos and Pacheco, 2017; Hudson et al., 2005). Combined with this, with the advancement of schooling, factors other than automaticity, such as general linguistic skills (semantic and syntactic awareness) and the ability to make appropriate inferences, assume a more relevant role in the adequate interpretation of a text (Schwanenflugel et al., 2006). In this sense, it is common for compensatory strategies to emerge over the years. In this way, students with dyslexia will seek to overcome these reading gaps by compensating for greater use of executive functions and working memory, in addition to other cognitive skills, such as lexical/semantic, processing speed, non-verbal IQ, short-term verbal memory and morphosyntax, start to play an important role in the development of reading skills (Peterson et al., 2013). Research with dyslexic students in more advanced educational segments verified normative levels in reading comprehension tests, revealing that this skill may be less impaired in adulthood with the emergence of compensatory mechanisms supported by other general cognitive abilities (Bazen et al., 2020; Faísca et al., 2023). Therefore, identifying students with dyslexia in more advanced years of basic education can represent a real challenge for Brazilian educational institutions, given that the country does not have systematized monitoring of reading in the academic context.

Furthermore, Dehaene et al. (2010) emphasized that the influence of culture and education could refine cortical organization since, based on imaging exams, they observed improvements in the processing of phonemes and in the visual responses of the fusiform and occipital cortex, even in late literate adults. The brain region related to visual word recognition is highly plastic, even in adult individuals, with rapid improvement in responses to reading stimuli. However, curiously, dyslexic children showed failures in this brain activation, a reading disorder that would be essentially phonological. Therefore, it is not yet possible to explain whether this failure is a cause or a consequence of the reading deficit (Dehaene and Cohen, 2011).

The main features of phonological processing deficits are poor phonological awareness, altered short-term verbal memory, and slow lexical retrieval. However, the phonological representations and grammatical processes may be intact, with a deficit elsewhere. Therefore, task requirements and, in particular, short-term memory overload seem fundamental in identifying phonological processing deficits. This scenario is clear in word reading tasks or longer stimuli, in which difficulties appear as the length of the sequence increases, and in most phonological awareness tasks that require the maintenance of segmented phonological units in short-term memory or require conscious access to these representations (Ramus and Szenkovits, 2008).

In addition to difficulties in decoding orthographic signs, phonological awareness and discrimination of sounds, dyslexic readers may present long-term verbal memory changes, with deficits in the formation of lexicon for storage, impacting the reading performance of irregular, infrequent and pseudowords (de Oliveira et al., 2021). In cognitive–linguistic terms, in this reading processing, word recognition (access to the mental lexicon) and understanding of what is read are essential. Two ways of recognizing the printed word during reading or two processes of accessing the mental lexicon are identified according to dual-route reading models (Coltheart et al., 2001). The dual-route model postulates that skilled reading can be accomplished by two separate pathways or routes, the lexical route, which relates reading to accessing a lexicon or memory store of previously seen written words, and a second route, the non-lexical route, which uses grapheme-phoneme correspondence rules (Coltheart et al., 2001).

Based on this model, Castles and Coltheart (1993) investigated the possibility of subtypes, indicating phonological developmental dyslexia and superficial developmental dyslexia, with impairments in the phonological and lexical routes, respectively. In reading through the lexical route, the visual presentation of a word activates the visual input lexicon, in which familiar words are stored, connecting to the meaning of the word. Therefore, the lexical route allows faster access to the semantic system than the phonological route, which first foresees a sequential decoding procedure, and only then accesses the meaning of the read material (Cunha et al., 2017; Salles and Parente, 2002). In this way, changes in the phonological route would affect the reading of unfamiliar words and pseudowords (phonological dyslexia). Inaccurate reading through the phonological route, with preservation of the lexical route, would characterize phonological dyslexia.

In contrast, impairments in the lexical route would make it difficult to read irregular words (visual or visual dyslexia). In this, he imprecise use of the lexical route, with preservation of the phonological route, would be characteristic of visual dyslexia, as well as the tendency to regularize irregular words during reading (Aurich et al., 2023). Dyslexia, characterized by changes in the lexical route, has received great attention from researchers due to its varied possibilities of manifestations. Changes in the input orthographic lexicon would affect reading aloud, lexical decision tasks, and homophone word comprehension; impairments in the output orthographic lexicon would make reading aloud and understanding homophones difficult; changes between the input orthographic lexicon and the output phonological lexicon would imply reading aloud; and finally, impairments in the phonological output lexicon would affect not only reading but also oral language tasks, such as verbal fluency and image naming (Friedmann and Lukov, 2008).

Nonetheless, the connectionist model presents some important reflection in relation to the dual route model for children with dyslexia (Snowling et al., 2020). The model clearly delineates word recognition and language comprehension as two components of reading. These elements should be viewed as interdependent, considering the sociocultural and educational context, which will favor a fluent and skilled reader. The connectionist framework infers that underlying reading skills interact with the environment. Such interactions are understood as complex, involving variations in more than one set of skills, at least in phonology and semantics, however plausibly also in visual (orthographic) skills (Snowling et al., 2020; Stuart et al., 2008). In this way, authors also highlight that decoding is related to reading ability, but also can be understand as a complex process, which implies mediating the meaning that the text communicates to the reader (Snowling et al., 2020). Some of these reading comprehension failures have been linked to decoding and meaning access failures, leading to poor performance in schoolchildren, as reported in the PISA assessments (INEP, 2021; OCDE, 2023).

In connectionist terms, dyslexia can be understood as a result of a failure in the mapping between orthography and phonology during reading acquisition. The authors state that this failure may be the result of the difficulty in creating reliable phonological representations and, consequently, creating the phonological stock. In the case of dyslexics, this could be observed in the low performance of non-word reading. Furthermore, the model indicates that this failure can be attributed to failures during the activation of the orthography-phonology connections, despite having normal representations, due to limitations in short-term verbal memory. Finally, the model also predicts that the phonological pathway may be compromised, not as a consequence of phonological deficits, but rather due to insufficiencies in the encoding of orthographic representations. In principle, this could occur as a result of a perceptual problem that prevented the system from analyzing letter sequences in the ideal way. From this perspective, Manis et al. (1996) proposed that phonological dyslexia could arise as a consequence of deficits at the level of phonological representations. In contrast, visual dyslexia may be due to a limitation of computational resources causing slow learning.

Nevertheless, despite strong evidence that changes in phonological processing characterize developmental dyslexia, many children with reading difficulties in irregular words present a correct reading of pseudowords. The attempt to differentiate the profiles of dyslexic individuals based only on reading patterns was not sufficient to identify distinct cognitive changes. In this context, neuroscience studies emerged relating visual processing disorders, with emphasis on visual attention (VA) disorder, to non-phonological reading profiles. In this way, it was understood that different reading patterns can result from dysfunctions in other brain areas (Peyrin et al., 2012). In light of this, Germano et al. (2014) analyzed the contribution of visual processing skills to reading Brazilian Portuguese. The authors also identified different cognitive subtypes of dyslexia among Brazilian students: those with a single cognitive change (phonological dyslexia or visual dyslexia), those with double changes (mixed profile dyslexia) and those without phonological or visual cognitive changes.

Additionally, psychophysiological studies highlight dyslexia as a visual, auditory and motor dysfunction with different subtypes, which can occur in other areas of the central nervous system. It is a complex disorder, as it may be associated with inadequate functioning of higher executive functions such as attention, phonological analysis, verbal-motor coordination, inhibition and feedback mechanisms, and memory. Dyslexic individuals would activate the right hemisphere more frequently during reading, evidence that may be related to compensatory mechanisms to improve performance (Chiarenza et al., 2014; Ramus et al., 2003).

Converging with this study, Medina and Guimarães (2021) suggest the inclusion of training in higher executive functions, such as cognitive flexibility, working memory, and inhibitory control, among others, in intervention programs with people with dyslexia to improve reading performance. The study findings positively and moderately correlated phonemic awareness and reading tasks with higher executive function skills.

In their study, Bazen et al. (2020) compared the reading and spelling skills of dyslexic adolescents, diagnosed early and late, with those of typical readers. Both groups of people with dyslexia had lower performance in pseudoword reading, spelling, phonemic awareness, rapid and automated naming, and visual attention. However, in the intragroup comparison of people with dyslexia, no differences were observed, which may suggest a late onset of reading gaps in some cases. The authors ruled out the possibility of greater severity of dyslexia between the groups, as the performance of reading skills and underlying cognitive skills was similar.

Faísca et al. (2023) conducted a study with dyslexic university students who speak European Portuguese. The findings revealed lower performance in reading-related skills in individuals with dyslexia, even with the systematic exposure to reading and writing provided by formal instruction. However, the test results highlighted two profiles of students with dyslexia. One of the groups presented a more attenuated phonological deficit associated with better performance in non-verbal IQ, vocabulary and working memory tests (verbal and visuospatial), which may suggest that general cognitive abilities help dyslexic individuals develop coping mechanisms and lifelong compensation associated with education and intervention programs.

In short, characterizing the reading processes in Brazilian pre-adolescent and adolescent students is essential so that possible gaps can be monitored, with dyslexic students identified and referred to therapeutic intervention programs. This study is based on the hypothesis that, by developing risk criteria for dyslexia, it will be possible to identify students at risk for reading learning disorder in our sample with the application of standardized screening instruments for this educational segment. Therefore, the aim of this study was to characterize the reading processes in adolescents and develop criteria to identify Brazilian students at risk for dyslexia in middle school.

## 2 Materials and methods

### 2.1 Participants

This study is observational and cross-sectional analytical, with a sample selected for convenience. The research was approved by the Research Ethics Committee, under Resolution nos. 5.924.651 and 5.989.860.

A total of 206 Brazilian students participated in the study, 53% male and 47% female, aged 11–15 years, regularly enrolled in middle school. The students were divided into four groups: 55 from 6th-grade students, 54 7th-grade students, GIII (46 8th-grade students), and GIV (51 9th-grade students). The parents and/or guardians who signed the Free and Informed Consent Form, and the students who signed a form agreeing to participate in the research were included. Students and/or guardians who refused to participate in the study were excluded from the sample.

The sample for the present study was collected in a public school in the city of Rio de Janeiro-RJ-Brazil. According to data from the institution’s Student Assistance Sector, among the students selected to receive student aid for 2023, 75% were black; 60% were residents of communities in Rio de Janeiro; 40% of those responsible were unemployed; and 56% were beneficiaries of government programs.

### 2.2 Experimental material

The Evaluation of the Reading Processes test (PROLEC-SE-R - middle school and high school) - were used to evaluate the reading of students participating in the study (Oliveira et al., 2022). The protocol consists of a screening version (collective) and a complete version (individual) in order to verify general reading ability in each school year (de Oliveira et al., 2020). As a procedure, initially, the students were subjected to the screening version (collective), and subsequently, the students who met the risk criteria for dyslexia were subjected to individual assessment. Next, we will describe the sequence of the procedures’ application.

#### 2.2.1 Collective application of the PROLEC-SE-R, screening version

In the first meeting, the screening version (collective) of PROLEC-SE-R was applied in the classroom. In its collective version, the protocol has six tests, being:

- *Lexical process tests*: composed of the Lexical Selection tests (LS – evaluates both the accuracy and speed of word recognition) and the Semantic Categorization test (SC – evaluates how quickly the student accesses the meaning of the words read).
- *Syntactic process tests*: composed of the Grammatical Structures Tests I (GSI – evaluates the student’s reading processing in sentences of greater grammatical complexity) and the Grammatical Judgment test (GJ – main objective of assessing how the student processes different syntactic structures in reading of a phrase).
- *Semantic process tests:* composed of the Expository Comprehension tests (EC – checks how the student extracts meaning from an expository text and integrates it into their memory) and the Narrative Comprehension test (NC – assesses not only the ability to extract the meaning of a narrative text, as well as the formation of a mental representation and the ability to make inferences, connecting the message of the text to previous prior knowledge).

After correcting each student’s response records, it was possible to determine scores for each student according to their performance in the PROLEC-SE-R tests. The scores followed the criteria described in the protocol. According to criteria described in the manual, classification was established for each process evaluated as normal reading ability – high (H), medium (M) or low (L), and presence of difficulties in reading ability – mild (D) or severe (DD), according to the school grade level. After the previously described scoring, new criteria were developed for this study, aiming to identify students at risk for dyslexia, as described below. To better understand the data collected, we also considered the performance of each student by reading process – lexical (LS + SC), syntactic (GSI + GJ) and semantic (EC + NC), always considering the worst performance in the tests related to each process. Therefore, if the student obtained a low performance (L) in a test of a certain process, and another indicative of difficulty (D) in tests of the same process, it was considered that there was difficulty (D) in that process.

#### 2.2.2 Risk criteria for dyslexia in the collective phase of the study, selection for the individual phase

**Figure 1:**
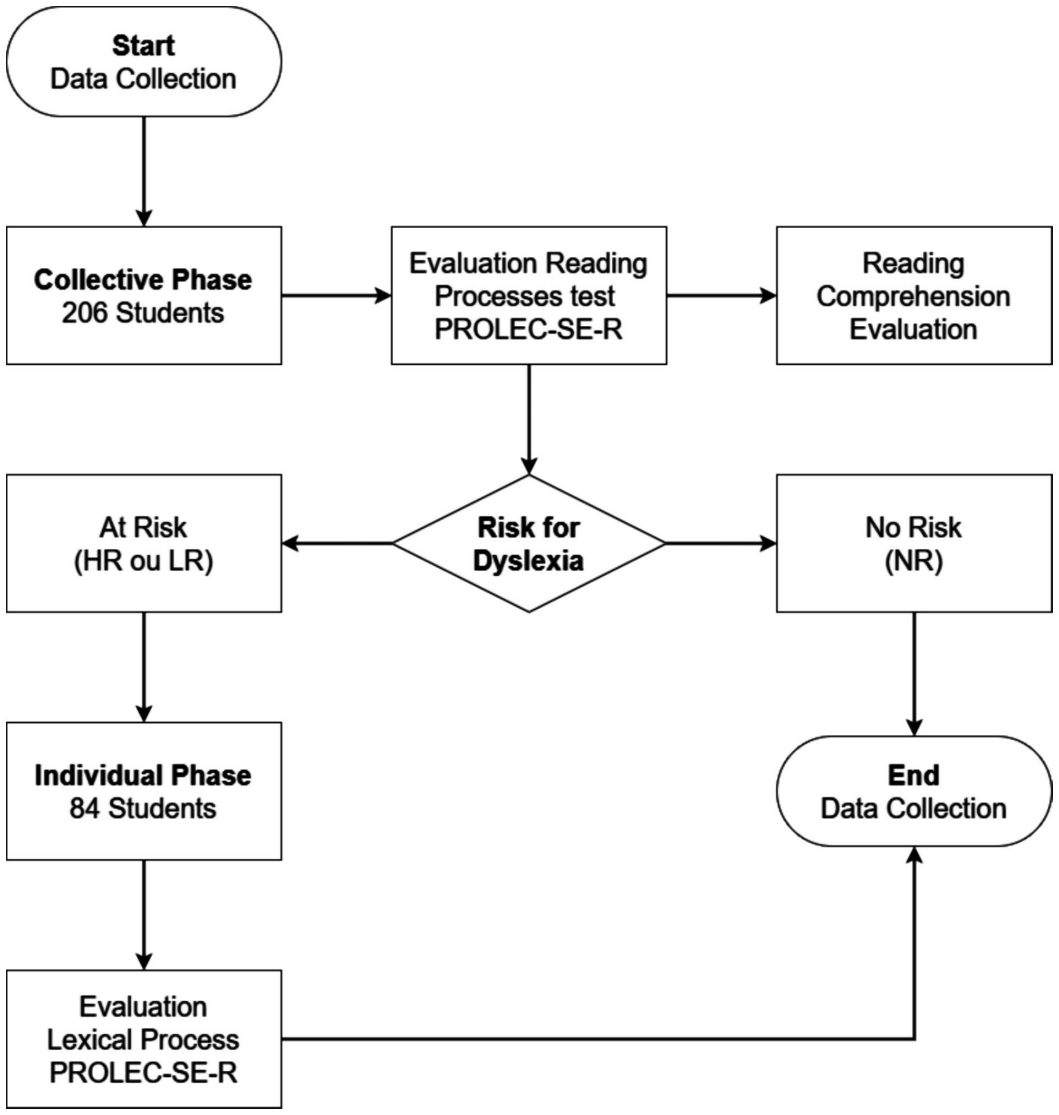
Study data collection flowchart. HR, High risk; LR, Low risk; NR, No risk.

Once the collective phase of the study was completed, the students were classified according to the risk for dyslexia prepared for this study. Based on the PROLEC-SE classification difficulties in reading ability – mild (D) or severe (DD), we developed a criterion to classify students according to their risk for dyslexia, as no risk (NR) and at risk (WR). Students who did not present difficulties (D or DD) in the PROLEC-SE-R reading processes were considered to be at no risk (NR); students with difficulties (D or DD) in just one reading process were considered to be at low risk (LR); and students with difficulties (D or DD) in two or more reading processes high risk (HR). Only students who presented low risk (LR) or high risk (HR) for dyslexia in the collective phase were assessed individually (Figure 1). The risk criterion for dyslexia was based on the literature, which indicates that weak word reading skills are enough to characterize students at risk of dyslexia, which should be further investigated and monitored in the school context (Ehri et al., 2001; Fletcher et al., 2019).

#### 2.2.3 Individual application of the PROLEC-SE-R reading process assessment tests – middle school and high school, lexical process tests of the full version

In this second meeting, students were assessed individually in an isolated room designated by the school management. Eighty-four students from 6th to 9th grade participated in the individual phase, divided into school groups, including 55 students (6th grade), 54 students (7th grade), 46 students (8th grade), and 51 students (9th grade).

Audio of the assessed students’ readings was recorded to assist in analyzing performance in the oral reading tests and checking possible errors. Only students classified as low risk (LR) or high risk (HR) were selected for the individual phase of the study were instructed to read a list of words (WR) and a list of pseudowords (PR) from the PROLEC-SE-R lexical process assessment, full version.

*Word reading (WR)—*The test consists of four lists with 24 words each, in which the student must read each one orally. The first and second lists are composed of high-frequency words, being short extension (SE) and long extension words (LE) words, respectively; the third and the fourth lists are composed of low-frequency, being short extension (SE) and long extension words (LE) words, respectively. With this test, we analyzed the functioning of the lexical and phonological routes in word recognition during student reading.

*Pseudoword reading (PR)–*In the same way as the previous test, the student must read two lists with 24 pseudowords each, the first being made up of short pseudowords (dissyllables) and the second list of long pseudowords (trisyllables and polysyllables). Its purpose is to examine the functioning of the phonological route in student reading.

The scores achieved by students in the *Word Reading (WR)* and *Pseudoword Reading (PR)* tests were subdivided into complementary scores, based on the raw score, the protocol proposes the classification of students’ performance for accuracy and speed in relation to the criteria of: (1) word frequency (high and low frequency); (2) reading of words (WR) and pseudowords (PR); (3) word extension (short and long). Regarding oral reading accuracy (A) they were classified as: normal reading ability (N) or low (L) reading ability; and also, as mild (D) or severe (DD) reading difficulties. Regarding speed (S), students were classified as having a normal reading ability as very fast (VF), fast (F) or medium (M), or reading difficulties – slow (SL) or very slow (VS).

#### 2.2.4 Risk criteria for dyslexia in the individual phase of the study

Once the individual phase of the study was completed, the oral reading performance data from the PROLEC-SE-R complementary scores were analyzed again. A new criterion was elaborated, considering the risk criteria developed previously. Students were classified as at risk (RR) or without risk (NR) for dyslexia. For this criterion, to be considered at risk for dyslexia (RR), we consider students classified by the protocol with reading difficulties mild (D) or severe (DD) in three or more subcategories in relation to accuracy: word frequency (HF-A, LF-A), reading of words (RW-A, PW-A), and word extension (SE-A, LE-A). We have also considerate students’ performance as slow (SL) or very slow (VS) in three or more subcategories in relation to speed: word frequency (HF-S, LF-S), reading of words (RW-S, PW-S), word extension (SE-S, LE-S). Students who did not present the accuracy and speed difficulties described previously were classified as without risk (NR). Finally, students classified as at risk (RR) in this phase were classified into visual (or “whole-word”), phonological and mixed, as proposed below. The terms adopted in this study were based on studies Coltheart et al. (2001) and Cestnick and Coltheart (1999). Coltheart and Jackson (1998) defined visual (or “whole-word”) dyslexia as a difficulty in orthographic word recognition, despite normal ability to map from letters to sound; and phonological dyslexia as a difficulty in phonological skills or to performe decoding processes for reading.

#### 2.2.5 Criteria for profiling students at risk for dyslexia

Finally, criteria were developed to identify different profiles of reading difficulties based on students’ performance in the PROLEC-SE-R accuracy and speed variables, in which three profiles were established among the students assessed individually:

a) Phonological profile (PP): students who had a performance classified as normal in reading high frequency words (HF-A/HF-S) but had difficulties reading pseudowords (PR-A/PR-S), low frequency words (LF-A/LF-S) and long extension words (LE-A/LE-S).
b) Visual profile (VP): students who had performance classified as normal in reading pseudowords (PR-A/PR-S) and who, however, had difficulties reading high frequency words (HF-A/HF-S) and short extension words (SE-A/SE-S).
c) Profile not defined (PND): students who presented heterogeneous performances in these subcategories, not meeting the criteria established for phonological and visual profiles.

#### 2.2.6 Statistical analyses

The data were statistically analyzed with the IBM SPSS Statistics program (Statistical Package for Social Sciences), aiming to characterize and compare performance in the described procedures, adopting a significance level of 5% (*p* < 0.05*), indicated by an asterisk. The Kruskal–Wallis tests were used to compare groups for all variables; the Mann–Whitney test when comparing groups when in a two-by-two situation; the Chi-Square Test when comparing categorical variables, and we performed the Two Proportions Z Test to analyze whether the proportion of responses for two certain variables and/or their levels is statistically significant.

## 3 Results

Tables 1, 2 demonstrated the results of the collective phase of the study, with the application of the screening version of PROLEC-SE-R, for raw score and classification, based on the application of the Kruskal–Wallis test and Chi-Square test, respectively.

**Table 1:** Raw score and classification of the population in the PROLEC-SE-R collective tests.

Processes	Variable	Group	Mean	Standard deviation	CI	*P*-value	Variable	Mean	Standard deviation	IC	*P*-value
Lexical process tests	LS	6th grade	38.3	8.8	2.3	<0.001*	SC	29.7	7.4	2	<0.001*
		7th grade	38.6	9.7	2.6			33.1	8.8	2.3	
		8th grade	43.5	7	2			41	11.2	3.2	
		9th grade	44.7	5.3	1.4			42	11	3	
Syntactic process tests	GSI	6th grade	12.1	3.1	0.8	<0.001*	GJ	9.7	3.3	0.9	<0.001*
		7th grade	13.2	3.7	1			11.9	4.2	1.1	
		8th grade	15.3	3.2	0.9			14.3	4.8	1.4	
		9th grade	16.3	3.1	0.8			17.5	5.7	1.6	
Semantic process tests	EC	6th grade	6.16	1.74	0.46	<0.001*	NC	5.62	1.88	0.5	0.016*
		7th grade	6.44	1.91	0.51			6.09	1.78	0.48	
		8th grade	7.13	1.87	0.54			6.28	1.8	0.52	
		9th grade	7.49	1.99	0.55			6.73	1.47	0.4	

CI, confidence interval; N, Number of students; LS, Lexical Selection; SC, Semantic Categorization; GSI, Grammatical Structures I; GJ, Grammatical Judgment; EC, Expository Comprehension; NC, Narrative Comprehension; H, High reading ability; M, Medium reading ability; L, Low reading ability; D, Mild reading difficulty; DD, Severe reading difficulty. *Kruskal–Wallis test (*p* < 0.05).

**Table 2:** Comparison of performance in the PROLEC-SE-R tests by school group.

Variable	Group	6th grade	7th grade	8th grade	Variable	6th grade	7th grade	8th grade
LS	7th grade	0.687			SC	0.039*		
	8th grade	0.001*	0.008*			<0.001*	<0.001*	
	9th grade	<0.001*	<0.001*	0.273		<0.001*	<0.001*	0.595
GSI	7th grade	0.086			GJ	0.001*		
	8th grade	<0.001*	0.012*			<0.001*	0.005*	
	9th grade	<0.001*	<0.001*	0.099		<0.001*	<0.001*	0.006*
EC	7th grade	0.448			NC	0.184		
	8th grade	0.002*	0.040*			0.07	0.561	
	9th grade	<0.001*	0.003*	0.39		0.001*	0.063	0.256

LS, Lexical Selection; SC, Semantic Categorization; GSI, Grammatical Structures I; GJ, Grammatical Judgment; EC, Expository Comprehension; NC, Narrative Comprehension. Mann–Whitney test *p < 0.05.

The results in Table 1 demonstrated that there was a significant difference between the groups for the lexical, syntactic and semantic processes tests for raw score. As there was a significant difference between grade level, the Mann–Whitney test was applied to compare the groups to their peers and thus accurately define the difference between them, as shown in Table 2.

In Table 2, we found a significant difference in the performance of the lexical process tests in almost all school groups, except for between 6th to 7th grade in LS and between 8th to 9th grade in LS and SC. In relation to the syntactic process tests of PROLEC-SE-R, GSI and GJ, the comparison of the groups was significant in almost all school years, except for the 6th to 7th grade and the 8th to 9th grade in GSI. In the semantic process tests, we again found a significant difference in most school groups compared to EC, with the exception of the 6th to 7th grade and the 8th to 9th grade. In the NC semantic process test, however, which required the interpretation of a narrative text with consultation, only the comparison of performance from the 6th to the 9th grade was significant.

Regarding classification, Table 3 presents the distribution of classifications proposed by PROLEC-SE-R among the school groups. Normal reading ability is represented by the classifications High (H), Medium (M) or Low (L), and reading difficulties are subdivided into Mild (D) or Severe (DD).

**Table 3:** Classification of the population in the PROLEC-SE-R collective tests.

Processes	Variables	Classification	6th grade	7th grade	8th grade	9th grade	Sample total	*P*-value
			*N* %	*N* %	*N* %	*N* %	*N* %	
Lexical process tests	LS	H	10 (18.2)	3(5.6)	1 (2.2)	5 (9.8)	19 (9.2)	0.001*
		M	33 (60)	26 (48.1)	36 (78.3)	37 (72.5)	132 (64.1)	
		L	7 (2.7)	9 (16.7)	3 (6.5)	5 (9.8)	24 (11.7)	
		D	5 (9.1)	6 (11.1)	4 (8.7)	3 (5.9)	18 (8.7)	
		DD	0 (0)	10 (18.5)	2 (4.3)	1 (2)	13 (6.3)	
	SC	H	5 (9.1)	4 (7.4)	4 (8.7)	3 (5.9)	16 (7.8)	0.205
		M	32 (58.2)	18 (33.3)	25 (54.3)	26 (51)	101 (49)	
		L	13 (23.6)	21 (38.9)	14 (30.4)	17 (33.3)	65 (31.6)	
		D	5 (9.1)	11 (20.4)	2 (4.3)	4 (7.8)	22 (10.7)	
		DD	0 (0)	0 (0)	1 (2.2)	1 (2)	2 (1)	
	LS + SC	Normal	48 (87.3)	35 (64.8)	38 (82.6)	43 (84.3)	164 (79.6)	0.006*
		D	7 (12.7)	9 (16.7)	6 (13)	6 (11.8)	28 (13.6)	
		DD	0 (0)	10 (18.5)	2 (4.3)	2 (3.9)	14 (6.8)	
Syntactic process tests	GSI	H	4 (7.3)	3 (5.6)	6 (13)	8 (15.7)	21 (10.2)	0.193
		M	33 (60)	26 (48.1)	21 (45.7)	30 (58.8)	110 (53.4)	
		L	7 (12.7)	11 (20.4)	13 (28.3)	7 (13.7)	38 (18.4)	
		D	8 (14.5)	11 (20.4)	6 (13)	6 (11.8)	31 (15)	
		DD	3 (5.5)	3 (5.6)	0 (0)	0 (0)	6 (2.9)	
	GJ	H	3 (5.4)	4 (7.4)	5 (10.9)	13 (25.5)	25 (12.1)	0.041*
		M	32 (58.2)	23 (42.6)	26 (56.5)	27 (52.9)	108 (52.4)	
		L	15 (27.3)	19 (35.2)	9 (19.6)	6 (11.8)	49 (23.8)	
		D	5 (9.1)	7 (13)	6 (13)	4 (7.8)	22 (10.7)	
		DD	0 (0)	1 (1.8)	0 (0)	1 (2)	2 (1)	
		Normal	41 (74.5)	35 (64.8)	35 (76.1)	44 (86.3)	155 (75.2)	
	GSI + GJ	D	11 (20)	16 (29.6)	11 (23.9)	6 (11.8)	44 (21.4)	0.172
		DD	3 (5.5)	3 (5.6)	0 (0)	1 (2)	7 (3.4)	
Semantic process tests	EC	H	12 (21.8)	16.7%	10 (21.7)	17 (33.3)	48 (23.3)	0.125
		M	25 (45.5)	50.0%	25 (54.3)	18 (35.3)	95 (46.1)	
		L	11 (20)	18.5%	5 (10.9)	12 (23.5)	38 (18.4)	
		D	6 (10.9)	14.8%	3 (6.5)	1 (2)	18 (8.7)	
		DD	1 (1.8)	0.0%	3 (6.5)	3 (5.9)	7 (3.4)	
	NC	H	17 (30.9)	22 (40.7)	13 (28.3)	16 (31.4)	68 (33)	0.451
		M	31 (56.4)	20 (37)	26 (56.5)	25 (49)	102 (49.5)	
		L	4 (7.3)	9 (16.7)	4 (8.7)	9 (17.6)	26 (12.6)	
		D	3 (5.5)	3 (5.6)	3 (6.5)	1 (2)	10 (4.9)	
		Normal	45 (81.8)	43 (79.6)	39 (84.8)	47 (92.1)	174 (84.5)	
	EC + NC	D	9 (16.4)	11 (20.4)	4 (8.7)	1 (2)	25 (12.1)	0.034*
		DD	1 (1.8)	0 (0)	3 (6.5)	3 (5.9)	7 (3.4)	

LS, Lexical Selection; SC, Semantic Categorization; GSI, Grammatical Structures I; GJ, Grammatical Judgment; EC, Expository Comprehension; NC, Narrative Comprehension. Chi-Square Test (*p < 0.05).

In relation to the performance classification, Table 3 shows the significant difference remained for the LS variable of lexical processes and GJ of syntactic processes. The results show that most students performed well in these processes. Once the collective phase of the study was completed, the students were classified according to the risk for dyslexia prepared for this study, that is, no risk (NR), low risk (LR); and high risk (HR) (Table 4), based on the application of the Kruskal–Wallis test.

**Table 4:** Distribution of school groups and the total sample according to risk for dyslexia.

Groups		GI (6th grade)		GII (7th grade)		GIII (8th grade)		GIV (9th grade)		Total		*P*-value
		*N*	%	*N*	%	*N*	%	*N*	%	*N*	%	
Risk for dyslexia	HR	7	12.7%	14	25.9%	6	13.0%	3	5.9%	30	14.6%	
	LR	15	27.3%	16	29.6%	11	23.9%	12	23.5%	54	26.2%	0.078
	NR	33	60.0%	24	44.4%	29	63.0%	36	70.6%	122	59.2%	

N, Number of participants; HR, High risk for dyslexia; LR, Low risk for dyslexia; NR, No Risk. Chi-Square Test (*p < 0.05).

The results in Table 4 did not indicate a significant difference between the school groups at risk for dyslexia. It was found that most students were not at risk for reading learning disorders for all groups analyzed. In relation to students who were at risk for dyslexia, 27.3% LR and 12.7% HR were observed for 6th grade; 29.6% LR and 25.9% HR for 7th grade; 23.9% LR and 13% HR for 8th grade; 23.5% LR and 5.9% HR for 9th grade.

Only students who presented low risk (LR) or high risk (HR) for dyslexia in the collective phase were assessed individually for reading words and pseudowords (Table 5), for raw and classification of performance, based on the application of the Kruskal–Wallis and Chi-Square test, respectively.

**Table 5:** Raw score and classification of the population in the PROLEC-SE-R individual reading tests.

Group	WR				PR			
	6th grade	7th grade	8th grade	9th grade	6th grade	7th grade	8th grade	9th grade
Mean	76.8	80.7	97.5	99.1	50.5	53.3	64.9	61.8
Standard deviation	15.1	21.8	21.8	14.1	12.5	18.4	19.5	14.5
CI	6.3	7.8	10.3	7.1	5.2	6.6	9.3	7.3
*P*-value	<0.001*				0.050*			
Classification	*N* %	*N* %	*N* %	*N* %	*N* %	*N* %	*N* %	*N* %
M	4 (18.2)	5 (16.7)	3 (17.6)	3 (20)	6 (27.3)	8 (26.7)	8 (47.1)	3 (20)
L	8 (36.4)	9 (30)	7 (41.2)	5 (33.3)	8 (36.4)	8 (26.7)	4 (23.5)	6 (40)
D	10 (45.5)	12 (40)	6 (35.3)	7 (46.7)	8 (36.4)	12 (40)	4 (23.5)	6 (40)
DD	0 (0)	4 (13.3)	1 (5.9)	0 (0)	0 (0)	2 (6.7)	1 (5.9)	0 (0)
*P*-value	0.761				0.664			

N, Number of students; WR, Word Reading; PR, Pseudoword Reading; M, Medium reading ability; L, Low reading ability; D, Mild reading difficulty; DD, Severe reading difficulty. *Kruskal–Wallis test (*p* < 0.05). **Chi-Square Test (*p* < 0.05).

In Table 5, the oral reading tests, applied only to students selected for the individual phase, we found a significant difference in most school years compared to WR and PR, however there was no significance for classification performance. As there was a significant difference between grade level, the Mann–Whitney test was applied to compare the groups to their peers and thus accurately define the difference between them, as shown in Table 6.

**Table 6:** Comparison of reading performance in the PROLEC-SE-R tests by school group.

Variable	Group	6th grade	7th grade	8th grade	Variable	6th grade	7th grade	8th grade
WR	7th grade	0.47			PR	0.597		
	8th grade	0.002*	0.025*			0.019*	0.057	
	9th grade	<0.001*	0.004*	0.91		0.029*	0.228	0.496

WR, Word Reading; PR, Pseudoword Reading. *Mann–Whitney test (*p* < 0.05).

It was possible to observe that, regarding reading performance, there was a significant difference in almost all school groups, except for the exception of the 6th to 7th grade and the 8th to 9th grade. Results demonstrate that there is a progression in reading performance as students’ level of education increases for reading words. However, in PR, only students from the 6th to the 8th and 9th grades showed a significant difference. There was no significance in the comparisons from the 7th grade to the 9th grade, suggesting that the students may have presented some difficulty in automating the phonological route. This lack of difference can be observed by the increase in students classified as Low reading ability and Mild reading difficulty, which may suggest learning difficulties or the presence of students at risk for reading disorders.

After analyzing the students’ performance in the oral reading tests, the students were again classified according to the criteria developed in this study for the individual phase, as students no risk (NR) and at risk (WR) for dyslexia. The Two Proportions Z test was used to analyze the distribution of students at risk in the variables accuracy and speed and compare them, as shown in Table 7.

**Table 7:** Distribution of risk for dyslexia in accuracy and speed of oral reading of words and pseudowords from the PROLEC-SE-R.

Risk	Accuracy			Speed			*p*-value
	*N*	%	*P*-value	*N*	%	*P*-value	
RR	16	19.0%	<0.001*	41	48.8%	0.758	<0.001*
NR	68	81.0%		43	51.2%		<0.001*

N, Number of participants; RR, With risk; NR, Without risk. *Two Proportions Z Test (*p* < 0.05).

A significant difference was observed regarding the risk classification for dyslexia in the accuracy variable, in which 19% of students assessed individually presented an inaccurate reading. In the speed variable, however, the risk groups for dyslexia did not show significant differences, with 48.8% of students showing slow reading. These results indicate that reading accuracy plays an important role in characterizing students at risk for dyslexia. Yet, reading speed did not allow us to separate students at risk from those without risk. Nevertheless, when analyzed together, in the intra-group comparison (*p*-value in the last column), the students at risk demonstrated that both accuracy and speed are important variables in characterizing students with reading process failures.

In this way, we carried out the comparison for the classifications with risk (RR) and without risk (WR) in order to verify whether the accuracy and speed could vary between them, according to Table 8. Table 8 shows the joint distribution of risk in accuracy and reading speed in students selected for the individual phase of the study.

**Table 8:** Distribution of risk for dyslexia in joint accuracy and speed (accuracy/speed) in the PROLEC-SE-R oral reading tests of words and pseudowords.

Risk A/S	*N*	%	*p*-value
RR/RR	13	15.5%	<0.001*
RR/NR	3	3.6%	<0.001*
NR/RR	28	33.3%	0.059
NR/NR	40	47.6%	Ref.

N, Number of participants; A, Accuracy; S, Speed; RR, With risk; NR, Without risk. *Two Proportions Z Test (*p* < 0.05).

The results indicate that there was a significant difference in the comparison of dyslexia risk criteria in the accuracy and speed variables. In a qualitative analysis, we found that the results indicated that 15.5% of the student presented deficits in both accuracy and speed; only 3.6% of the students who failed in accuracy reading had an adequate reading speed; and that 33.3% of the students evaluated had a sufficient accuracy but slow reading (speed). It was also found that 52.4% of students met risk criteria in the accuracy and/or speed variables (RR/RR and NR/RR) and that 47.6% (NR/NR), in fact, did not present risks for dyslexia.

In the search to better characterize this population at risk, we also carried out another analysis to identify different profiles phonological profile (PP), visual profile (VP) and undefined profile (PND) among the reading difficulties, related to accuracy and speed variables (Table 9).

**Table 9:** Profile distribution of reading difficulties in accuracy and speed in the PROLEC-SE-R oral reading tests of words and pseudowords.

Profile	Accuracy			Speed			*P*-value
	*N*	%	*P*-value	*N*	%	*P*-value	
PP	15	17.9%	<0.001*	18	21.4%	<0.001*	0.560
VP	5	6.0%	<0.001*	18	21.4%	<0.001*	0.004*
PND	64	76.2%	Ref.	48	57.1%	Ref.	0.009*

N, Number of participants; PP, Phonological profile; VP, Visual profile; PND, Profile not defined. *Two Proportions Z Test (*p* < 0.05).

The results of Table 9 indicated that there was a significant difference between the phonological, visual and undefined profiles for both accuracy and speed. These results suggest a difference in the pre-determined reading performance of students at risk for dyslexia. Among the total number of students assessed individually, Table 9 showed that regarding the phonological profile, the results indicated that accuracy and speed characterized 17.9 and 21.4% of at-risk students, respectively. However, when analyzed together, in the intra-group comparison (*p*-value in the last column), did not indicate a significant difference. Neither accuracy nor speed were significant, indicating that the phonological deficit impairs accuracy and speed performance, that is, failures in performing grapheme-phoneme decoding impact the accurate recognition of words and, consequently, impair reading speed.

Considering the visual profile, accuracy had a smaller impact (6%) and speed also affected 21.4% of at-risk students. Yet, when analyzed together, in the intra-group comparison (*p*-value in the last column), indicate a significant difference. This finding allows us to infer that students with the visual subtype have less difficulty in visually recognizing words, especially those that can be read by decoding the direct grapheme-phoneme relationship but may fail when there is some irregularity. Thus, failures with speed may be related to irregularities present in some words read. Therefore, this type of profile may be difficult to identify in the school context, as mild difficulties in accuracy may not be perceived as a reading failure.

We also highlight that the rate of students with an undefined profile reduced from 76.2 to 57.1% in the speed category, observing a significant difference when comparing the variables. The undefined profile draws our attention to the fact that there are students with reading difficulties, but who do not fit into the category of phonological or visual deficits. This profile draws our attention because these students may have reading difficulties resulting from deficits in the learning processes, such as methodological flaws, but which deserve further investigation. Hence, in Table 10, we compared the phonological, visual and undefined profiles to verify and refine whether there would be any discrepancy from the previous classification.

**Table 10:** Profile distribution of reading difficulties in joint accuracy and speed (accuracy/speed) in the PROLEC-SE-R oral reading tests of words and pseudowords.

Profile A/S	*N*	%	*P*-value
PP / PP	2	2.4%	<0.001*
PP / PND	12	14.3%	<0.001*
PP / VP	1	1.2%	<0.001*
PND / PP	15	17.9%	0.002*
PND / PND	33	39.3%	Ref.
PND / VP	16	19.0%	0.004*
VP / PP	1	1.2%	<0.001*
VP / PND	3	3.6%	<0.001*
VP/VP	1	1.2%	<0.001*

*N* = Number of participants; A, Accuracy; S, Speed; PP, Phonological profile; VP, Visual profile; PND, Profile not defined. *Two Proportions Z Test (*p* < 0.05).

Table 10 presents the joint distribution of reading accuracy and speed profiles in students selected for the individual phase of the study.

Table 10 indicates that there was a difference between accuracy and speed for the profiles investigated. When observing the undefined profile, when the comparison occurs, we note that only 19% (PND/PND) remained in this classification, while the remainder were redefined as phonological (34.2%, PND/PP; PP/PND) or visual (22.6%, PND/VP; VP/PND). We noted a reduced number of students classified as “pure” profiles, that is, with flaws in accuracy and speed resulting from phonological or visual (lexical) flaws, with 2.4% having a pure phonological profile (PP/PP) and 1.2% having a “pure” visual profile (VP/VP). Thus, analyzed together, 34.6% met the criteria for phonological profile, 23.8% for visual profile, and 2.4% for mixed profile (PP/VP or VP/PP).

Finally, in Table 11, we use the Chi-Square test to relate the risk distributions for risk of dyslexia with the profiles obtained according to the students’ performance in the variables of accuracy and speed of oral reading of words and pseudowords from the PROLEC-SE-R.

**Table 11:** Relationship between risk classification for dyslexia and profile, in accuracy and speed, in the PROLEC-SE-R oral word and pseudoword reading tests.

Variables		PP		VP		*P*-value
		*N*	%	*N*	%	
Accuracy	WR	7	46.7%	2	40.0%	0.795
	NR	8	53.3%	3	60.0%	
Speed	WR	10	55.6%	10	55.6%	1.000
	NR	8	44.4%	8	44.4%	

N, Number of participants; RR, With risk; NR, Without risk; PP, Phonological profile; VP, Visual profile.

In Table 11, we found that there was no significant difference between the phonological and visual profiles in relation to the risk regarding accuracy and speed. A total of 46.7% of students classified with a phonological profile and 40% of those considered with a visual profile presenting a risk for dyslexia in terms of accuracy. When considering oral reading speed, 55.6% of students classified with a phonological and visual profile were at risk for reading learning disorders. These findings suggest that both accuracy and speed criteria are important for indicating risk for dyslexia, but with different impacts observed for each profile.

## 4 Discussion

This study aimed to characterize the reading processes in adolescents and develop criteria to identify Brazilian students at risk for dyslexia in middle school. The analysis of the descriptive results of the population in the collective reading process tests of the PROLEC-SE-R revealed a significant difference in most groups, suggesting an improvement in reading processes during middle school. Systematized reading practices in formal instruction are fundamental in improving reading skills (Menard and Wilson, 2014; Rosendo et al., 2023) since learning to read is a growing process that can extend beyond 18 years of age (Fernandes et al., 2015).

The results of the study showed a significant difference in the performance of collective lexical process tests in almost all school groups, except for the 6th to the 7th grade in the LS test (Lexical Selection) and from the 8th to the 9th grade in SL and SC (Semantic Categorization). The results suggest an evolution in reading fluency in students in middle school since, in all tests of the lexical process, the scores in the 8th and 9th grades were higher than those achieved in the 6th grade. In their study, Bigozzi et al. (2017) emphasize the importance of reading fluency in the learning of middle school and high school students. Reading fluency can also be an important predictor of educational level (Fernandez and Arriondo, 2021).

However, the analysis of the groups’ performance data in the lexical process tests suggests that, possibly, the evolution of reading fluency in middle school tends to be slower than in elementary school since students in the 6th and 7th grades and the 8th and 9th grades, obtained similar scores in some tests. Studies with Brazilian middle school students reveal a gradual increase in reading speed and accuracy in this segment, with a tendency for parameters to stabilize in the final years (Alves et al., 2021; de Andrade et al., 2019; Pires et al., 2024).

In relation to the syntactic process tests of PROLEC-SE-R, GSI (Grammatical Structure I) and GJ (Grammatical Judgment), the comparison of the groups proved to be significant in almost all school years, with the exception of the 6th to the 7th grade, and from the 8th to the 9th grade in GSI. The data corroborate the study by Capovilla et al. (2004), which revealed a significant effect of schooling on the syntactic awareness of elementary school students. Efficient reading requires the development of metalinguistic skills, such as phonological awareness, syntactic awareness, pragmatic awareness and metatextual awareness. The development of these skills is essential so that more complex levels of reading ability can be achieved, such as reading comprehension (da Silva and da Fonseca, 2021).

In the semantic process tests, we again found a significant difference in most school groups compared to EC (Expository Comprehension), except for the 6th to the 7th grade and the 8th to the 9th grade. In the NC (Narrative Comprehension) semantic process test, however, only the comparison of performance from the 6th to the 9th grade was significant. Reading comprehension involves connecting information from a written text with prior knowledge of a given subject and requires not only basic-level reading processes but also distinct high-level processes, such as inferential capacity and monitoring of what is being understood (Andrade and Dias, 2006).

The different textual genres and the different cognitive skills required by EC and NC may have contributed to the differences in performance observed in school groups in the semantic process tests. In their study, Barth et al. (2014) report that narrative structures tend to be read with greater fluidity than expository structures. Furthermore, textual genres require different cognitive processing as they differ in structure, characteristics, grammar and social action.

Narrative texts present a sequence of events based on temporality and causality, with a more frequent vocabulary. In contrast, expository texts reveal information on a given subject in a logical order of representation, with a more technical vocabulary, and generally require greater prior knowledge so that understanding can be achieved. Therefore, understanding narrative texts tends to be simpler than expository texts (Morini and Capellini, 2023). Our findings also agree with Oliveira and Germano (2025). Oliveira and Germano (2025) analyzed the textual genres of elementary school textbooks (1st to 5th grade). The authors found that there is a predominance of didactic focus on narrative texts, and less focus on expository texts. The authors highlight that instead of enabling progress in reading skills in other textual genres, in addition to narrative, schools end up exploring and presenting, most of the time, extremely simplified, non-authentic texts. Contrary to the assumptions of the educational guidelines for the Portuguese language, expository texts are not very accessible in elementary school Portuguese textbooks. Thus, the findings of this study agree with Oliveira and Germano (2025) and end up demonstrating that, due to this focus on elementary school education, middle school students may still have difficulty reading expository texts.

In the collective evaluation of the reading processes of this sample, our findings indicated that most students had performance classified as normal, being classified as not at risk for reading learning disorders, according to the criteria developed for this study. However, our results also showed that many students from all school years needed help in their reading processes, suggesting low proficiency in their lexical, syntactic and semantic access.

When analyzing the distributions of the classifications proposed by PROLEC-SE-R among the school groups, we observed significant differences only in the LS (Lexical Selection) and GJ (Grammatic Judgment) tests. In the rest of the tests, the groups maintained similar distributions in relation to the reading performance, which was considered normal, with the presence of difficulties. However, when grouping the tests according to the process evaluated, both in the lexical process (Lexical Selection + Semantic Categorization) and in the semantic process (Expository Comprehension + Narrative Comprehension), we observed significant differences in the distribution of classifications in relation to reading ability, among school groups. The performance of the school groups in the syntactic process tests (Grammatical Structure I and Grammatical Judgment) was also relevant data in our study, as even though no significant difference was found in the distributions of PROLEC-SE-R classifications in relation to reading ability, this was the process in which the school groups presented the most difficulties. The syntactic process is a fundamental metalinguistic skill in the development of reading, whose improvement occurs mainly in formal instruction.

Stuart et al. (2008) highlight that reading words is a prerequisite for understanding texts. Thus, although phonetics and word recognition. Furthermore, the semantic context and grammatical knowledge contribute to the reading of isolated words, but their greatest influence is on the understanding of the text (Nation and Norbury, 2005). Studies highlight that a proficient reader would be the result of a combination of good performance in visual word recognition or the decoding process based on phonetics, combined with world knowledge. Thus, we note that some students failed to automate these processes, and failed to perform reading comprehension of expository texts, which require greater abstractions (Nation and Norbury, 2005; Stuart et al., 2008).

These difficulties in the reading processes presented by the students evaluated collectively may also be related to many factors, such as the socioeconomic indexes of the sample, the pandemic context itself, or even motivational issues for carrying out the collective tests. In addition, according to Verwimp et al. (2023), families with low socioeconomic status or little educational background tend to spend less shared reading time with their children or have fewer books at home, affecting early reading development.

In Brazil, 92% of basic education schools adopted hybrid or remote teaching in the pandemic context (INEP, 2021). Remote teaching appears to have been especially challenging for students with a low socioeconomic level due to difficulties in accessing technological resources and educational materials necessary for this modality (Barbosa et al., 2022). Rosendo et al. (2023) evaluated fluctuations in reading fluency parameters in Portuguese students in the 3rd grade of elementary school in the pandemic context. The results of the study showed a performance in reading fluency and accuracy below expectations for low-income students, beneficiaries of government financial aid programs. In their study, the authors also report the importance of stimulating students’ reading skills during periods when they are away from school, with reading practices encouraged by their families. However, according to Santana et al. (2022), the reading difficulties of Brazilian students cannot be attributed exclusively to the remote teaching practiced during the COVID-19 pandemic since Brazilian studies prior to the pandemic context already showed lags in predictive abilities of reading in students of elementary school, mainly in relation to aspects of perception of sounds and syllables and letter–sound conversion.

The results of PISA 2018, prior to the pandemic, already showed low reading performance among Brazilian students, who in the assessment achieved 413 points in this skill, lower than the OECD average of 487 in 2018 (OCDE, 2023). After four years and a turbulent pandemic period in Brazilian educational institutions, in PISA 2022, our students achieved an average performance of 410 points in the assessment (OCDE, 2023).

Consistent with Oliveira et al. (2022), in approximately 40% of PROLEC-SE-R collective screenings, mild (D) or severe (DD) difficulties may arise in the reading processes. Therefore, it is recommended that individual tests of the protocol be applied for a more in-depth assessment of the reading processes. Our findings showed that students had difficulties in reading words and pseudowords performance. Therefore, aiming to differentiate possible reading learning disorders from other changes in reading processes resulting from various factors, we individually evaluated part of our sample, as suggested by the protocol used in the study. In the distribution of risk for a reading learning disorder, our results from the collective phase revealed 27.3% of students at low risk (LR) and 12.7% at high risk (HR) for dyslexia in GI (6th grade), 29.6% with LR and 25.9% with HR in GII (7th grade); 23.9% with LR and 13% with HR in GIII (8th grade); 23.5% with LR and 5.9% with HR in GIV (9th grade). Taking together, regarding the risk classification for dyslexia in the accuracy variable, 19% of students assessed individually presented an inaccurate reading. However, for speed variable, 48.8% of students showed slow reading.

As schooling progresses, words should be read more quickly, as long as the student has formed in his memory a good recognition of visual words and a good grapheme-phoneme decoding ability, which will ensure good reading fluency and successful comprehension of the text (Stuart et al., 2008). These findings corroborate the results of this study, which shows that there was an evolution in the word reading test, with the progression of the students’ performance. However, when observing the performance classification for word reading, it was noted that many students performed below the reading ability level and with difficulty, indicating that the process of decoding or accessing the orthographic lexicon was not fully learned by the adolescent students. Furthermore, the results indicated that there was no such progression when observing the reading of pseudowords, which also had poor performances, which suggests reading failures through the phonological route, that is, there was a failure in the grapheme-phoneme decoding process. These difficulties might suggest reading disorder, such as dyslexia.

Dyslexia is a neurobiological, multifactorial disorder that manifests itself early, even in the preschool phase. It can be comorbid with language disorders, attention and motor coordination problems, as well as emotional and behavioral difficulties (Snowling et al., 2020). Students with dyslexia may present academic skills below those expected for their chronological age, with possible losses in school performance, even in the presence of adequate educational instruction (APA, 2014).

The DSM-V (APA, 2014) describes dyslexia as a specific learning disorder characterized by persistent difficulties in reading ability, such as inefficient letter/sound decoding and slow and imprecise reading. Dyslexic readers may also present long-term verbal memory changes, impacting the reading performance of irregular, infrequent words and pseudowords (de Oliveira et al., 2021).

In their study, Mantovani et al. (2021) compared students without learning complaints with students diagnosed with dyslexia. The results revealed a lower performance of students with dyslexia in all PROLEC tests. de Oliveira et al. (2012) evaluated students with dyslexia, students with learning disorders and typical students. Students from elementary school with dyslexia and learning disabilities performed worse than typical students in the PROLEC tests, with people with dyslexia performing better than students with learning disabilities.

In the individual phase, the study findings revealed influence of speed and accuracy for risk identification, but reading speed made it possible to differentiate students at risk from those without risk. But, when analyzed together, students at risk had both accuracy and speed difficulties, being an important variable to characterize students with reading process failures from those without reading difficulties in educational context. These findings agree with the diagnostic criteria described in the DSM-5 (APA, 2014), that emphasize that alteration in accuracy and speed (fluency) can be related with dyslexia.

Fernandes et al. (2015) highlighted reading speed as a fundamental measure in identifying students with reading learning disorders, more important than reading accuracy in more advanced educational segments. In line with this study, Bigozzi et al. (2017) emphasized reading speed as an important predictor of academic performance, being more relevant than reading accuracy.

When analyzing the profiles of students at risk for dyslexia, regarding the phonological profile, the results indicated that accuracy and speed characterized 17.9 and 21.4% of at-risk students, respectively. However, when analyzed together, either accuracy nor speed were significant, indicating that the phonological deficit impairs accuracy and speed performance, that is, failures in performing grapheme-phoneme decoding impact the accurate recognition of words and, consequently, impair reading speed. These findings suggest that both accuracy and speed criteria are important for indicating risk for dyslexia in Brazilian Portuguese. In agreement, Snowling et al. (2020) also point out that students with persistent fluency deficits should be treated with caution, since reading problems observed in literacy can persist into adulthood.

In relation to reading, the dual route theory postulates two different ways in which a skilled reader can pronounce a written word (Coltheart et al., 2001). The authors state that reading may occur via the lexical route, when there are words that can be recognized directly by accessing the representation of their orthographic form in an internal lexicon (lexical reading). The lexical route will successfully process all words that a reader is familiar with but will not recognize unfamiliar words or non-words. Instead, reading via the phonological route, which is an indirect mechanism that requires grapheme-phoneme conversion, used for unknown words, will correctly sound out non-words, and regular words that follow typical grapheme–phoneme correspondence or pseudowords.

In this way, changes in the phonological route would affect the reading of unfamiliar words and pseudowords (phonological dyslexia). On the other hand, impairments in the lexical route would make it difficult to read irregular words (visual dyslexia). Inaccurate reading through the phonological route, with preservation of the lexical route, would characterize phonological dyslexia. In contrast, the imprecise use of the lexical route, with conservation of the phonological route, would be characteristic of visual dyslexia, as well as the tendency to regularize irregular words during reading (Aurich et al., 2023). According to the dual-route model (Coltheart et al., 2001), the students in this study presented failures in reading words and pseudowords, classified as a phonological profile, characterized by failure in precision and speed, suggesting that failures in performing grapheme-phoneme decoding impact on the accurate recognition of words and, consequently, harm reading speed.

Cohen et al. (2008) analyzed cortical activations during reading using imaging tests. The findings showed an effect of word length on reading latencies, reflecting serial reading strategies. Therefore, longer words tend to be read via the phonological route. Shaywitz et al. (2017) analyzed neural activation patterns during reading in typical and dyslexic children, revealing the presence of a persistent phonological deficit. In this way, older dyslexic readers would begin to read familiar words by memorization (lexical route), maintaining reading difficulties in unfamiliar words. However, phonological difficulties are neither necessary nor sufficient to explain dyslexia (Pennington, 1991). Although poor phonology is the impairment most consistently and traditionally associated with dyslexia, many children at familial risk for dyslexia who do not succumb to reading difficulties may have deficits outside the phonological domain and also have problems with phonological awareness (Snowling and Melby-Lervåg, 2016).

Considering the visual profile, accuracy had a smaller impact (6%) and speed also affected 21.4% of at-risk students. Yet, when analyzed together, visual profile was impacted for both accuracy and speed. This finding allows us to infer that students with the visual subtype have less difficulty in visually recognizing words, especially those that can be read by decoding the direct grapheme-phoneme relationship but may fail when there is some irregularity. In cognitive-linguistic terms, word recognition (access to the mental lexicon) and understanding of what is read are essential in reading processing. The lexical route, in which familiar words are stored, allows faster access to the semantic system than the phonological route, which first foresees a sequential decoding procedure, and only then accesses the meaning of the read material (Cunha et al., 2017; Salles and Parente, 2002).

Despite strong evidence that changes in phonological processing characterize developmental dyslexia, many children with reading difficulties in irregular words showed intact reading of pseudowords (Peyrin et al., 2012). Castles and Friedmann (2014) highlight that the different manifestations of dyslexia (subtypes) could be difficult to identify in very regular languages since words can generally be read correctly through grapheme–phoneme conversion, hindering the identification of deficiencies in the lexical reading route.

The orthographic transparency of the language also influences the development of reading, and the difficulties associated with this skill. This finding may be related to the fact that Brazilian Portuguese presents many words with direct and univocal grapheme-phoneme conversion, which allows the formation of long-term lexical memory, which favors reading with greater precision (Scliar-Cabral, 2003). Furthermore, the students in this study have already gone through the literacy process and should have better performance in reading words (Scliar-Cabral, 2003; Oliveira et al., 2016). Therefore, the perception of difficulties in reading words reinforces the possibility of specific profiles, such as visual. Thus, failures in accuracy and speed of words, but not in pseudowords, should be viewed with caution, as the linguistic context itself enables the formation and use of reading via the lexical route, especially for adolescent students.

Hence, failures with speed may be related to irregularities present in some words read. Therefore, this type of profile may be difficult to identify in the school context, as mild difficulties in accuracy may not be perceived as a reading failure. Our findings are in agreement with Germano et al. (2014), who identified different cognitive subtypes of dyslexia among Brazilian students evaluated in their study: those with a single cognitive alteration (phonological or visual), those with double alteration (mixed profile) and those no phonological or visual cognitive changes.

We also highlight that 39.3% of students had an undefined profile, which draws our attention to the fact that there are students with reading difficulties, but who do not fit into the category of phonological or visual deficits. This profile draws our attention because these students may have reading difficulties resulting from deficits in the learning processes, such as methodological flaws, but which deserve further investigation. Psychophysiological studies have highlighted dyslexia as a visual, auditory and motor dysfunction with different subtypes, which can occur in various areas of the CNS (Chiarenza et al., 2014). In this sense, it is important to inform that these students who met the risk criteria for dyslexia in the individual phase of the study and also fit into the established profiles of reading difficulties represented 14.1% of the total sample of this study. The study by Bassôa et al. (2021) provides relevant information about the estimated number of individuals with dyslexia, which would be around 5–10% of the world population and 7% of the Brazilian population. Other studies reveal that dyslexia can affect between 3 and 10% of school-age children, and in Brazil, it can affect 5–15% of students (Mantovani et al., 2021).

However, Snowling et al. (2020) report that the dual-route model may present some limitations, especially when a word is irregular, and the two pathways (lexical and phonological) generate information that conflicts. Resolving this conflict may take time, impairing recognition and reading speed. In the case of our study, the profiles allude to impaired speed and accuracy, which should be considered as important variables to characterize students at risk for dyslexia, regardless of the profiles reported here. In this way, we can understand that our findings agree with Pennington (1991), that emphasizes that dyslexia is the result of multiple risks that accumulate to explain these reading failures.

Another finding of this study refers to the “pure” profiles, with 2.4% presenting a pure phonological profile and 1.2% presenting a “pure” visual profile. Still in relation to the undefined profiles, it was also possible to note that there was a predominance of failures, thus being redefined as phonological (34.2%) and visual (22.6%).

Taken together, the findings of this study corroborate Snowling et al. (2020), who highlight that a child’s score on a standardized word reading test reflects multiple sources of variation (and difficulty). For the authors, part of the complexity associated with dyslexia arises because the predominant proximal cause – a phonological deficit – is often not the only deficit observed. The results of this study demonstrated that, despite the different profiles, only a small portion of the students (1.2% %) were classified as having a visual profile. However, this finding gives us an idea of the defective functioning of reading execution and does not allow us to eliminate a basic phonological deficit, since in order to read, the student will resort to the phoneme in its conversion. This finding also agrees with the results of Valdois (2022), who in her theory of the visual-attentional deficit highlights that access to the phoneme is still necessary for reading.

As explained in our methodology, all students who presented the Free and Informed Assent Form and the Free and Informed Consent Form were included in our sample, as this is a study characterizing the reading processes of middle school adolescents. However, our findings showed that, of the students assessed individually for being at risk for dyslexia, only 13% had records of interdisciplinary diagnoses known to the educational institution. Considering students with interdisciplinary reports, we found that 27.3% diagnosed with dyslexia met the study’s risk criteria, presenting a phonological profile (PP) or mixed (PP/VP); 27.3% with reports relating to other cognitive–linguistic disorders met the risk criteria for dyslexia, but were classified as having an undefined profile (PND); 45.4% diagnosed with ADHD did not meet the study’s risk criteria for dyslexia and also presented an undefined profile (PND).

Since 52.4% of students met the risk criteria for reading learning disorder in the individual phase, only 13% had interdisciplinary diagnoses; this study highlights the importance of monitoring reading ability in the final years of elementary school. In Brazil, underdiagnosis and late diagnosis are frequent, requiring monitoring and identification instruments for reading learning disorders in all segments of basic education (Bassôa et al., 2021).

The pandemic context may indeed have heightened this reality since, like schools, health services were also affected by COVID-19. Furthermore, the remote teaching practiced by educational institutions during this period may have made it difficult for teachers to identify students with reading difficulties, reducing referrals to health services. During the COVID-19 pandemic, many Brazilian teachers suffered from limitations in technological resources, lack of training to practice remote teaching, and difficulties in communicating online with students (Starling-Alves et al., 2023).

Another hypothesis for this finding lies in the possibility that, in a second segment, many students may manifest compensatory mechanisms to deal with their reading gaps, making it difficult for teachers to identify dyslexia. Research with dyslexic students in more advanced educational segments verified normative levels in reading comprehension tests, revealing that this skill may be less impaired in adulthood with the emergence of compensatory mechanisms supported by other general cognitive abilities (Bazen et al., 2020; Faísca et al., 2023).

Snowling (2008) also proposed to characterize the phenotypic cognitive profile of dyslexics. The author proposed to characterize students in relation to the composite of tests that assess aspects of phonology, visuospatial and attention. As results, the author stated that the findings challenge the notion that dyslexia is caused by a specific phonological deficit. Instead, the author suggests that there are more diffuse difficulties in the risk group with literacy impairment, or that some dyslexics were able to compensate for their difficulties. The author agrees that her results present a complex picture, but that it probably reflects the varied developmental trajectories observed in any developmental disorder. In addition, the author also indicated that, although it is possible, at the group level, to identify risk factors related to reading difficulties.

Thus, the findings of this study agree with (Snowling, 1998, 2008). In these studies, the author reflects that, normally, the reading failures of dyslexics are compatible with phonological processing problems. However, they suggest that the severity of a child’s phonological difficulty can affect the way in which their reading system is configured, being influenced by the type of teaching, but in any case, both “phonological” and “visual” dyslexics will have impairments in their school performance.

Besides, neurological approaches refer that dyslexic individuals would activate the right hemisphere more frequently during reading, evidence that may be related to compensatory mechanisms to improve performance (Chiarenza et al., 2014). Also, Dehaene et al. (2010) revealed that the influence of culture and education can refine cortical organization. However, dyslexic children presented failures in this brain activation, a reading disorder that would be essentially phonological (Dehaene and Cohen, 2011). In this sense, it is common for compensatory strategies to emerge to overcome these reading gaps. Other cognitive skills can play an important role in the development of reading skills (Peterson et al., 2013). Converging with this study, Medina and Guimarães (2021) suggest the inclusion of training in higher executive functions, such as cognitive flexibility, working memory, and inhibitory control, among others, in intervention programs with people with dyslexia to improve reading performance.

The limitation of this study is the fact that our sample was collected in a single educational institution, despite Brazil being a country with multiple educational contexts. We suggest that, in subsequent studies, the risk criteria for dyslexia developed in this study be applied in other regions of Brazil, with students from public and private institutions understanding that socioeconomic–cultural factors permeate the adequate development of reading skills.

We also emphasize that only oral reading tests were administered individually to students classified as at risk for dyslexia in our collective phase. However, understanding that dyslexia is a heterogeneous reading disorder with different manifestations, we recommend that standardized tests to assess visual skills and other cognitive–linguistic skills be carried out to identify individuals with reading learning disorder with different subtypes of dyslexia.

## 5 Conclusion

From this study, we verified that the majority of students evaluated collectively achieved performance in reading processes classified as normal. However, our results also showed that many students from all school years showed reading gaps, suggesting low proficiency in their lexical, syntactic and semantic access.

The risk criteria for dyslexia in this study, drawn up based on the students’ performance in the PROLEC-SE-R reading process tests, proved to be effective. We showed that all students in our sample, whose report of reading learning disorder was known institutionally were identified in the individual phase of the study as being at risk for dyslexia and meeting the proposed criteria for phonological or mixed profiles. However, the risk criteria developed in this study must be applied in other educational contexts, with students from public and private institutions, to actually verify their sensitivity.

The criteria developed to identify different profiles of reading difficulties highlighted students with a phonological profile, visual profile and mixed profile (phonological and visual) in accordance with Brazilian and international literature. We also emphasize that the proposed criteria for identifying different reading difficulty profiles can favor clinical reasoning in the investigation of dyslexia subtypes, a literature that is still scarce in Brazil.

Our findings also revealed that many students in our sample with significant reading gaps did not have any pedagogical support or guidance for specific assessments in the health network. In this way, this study raises awareness of the importance of monitoring reading skills in middle school in the educational context so that students at risk for dyslexia and other learning disorders, often associated with reading gaps, are identified.

Finally, we highlight the need for more scientific research with pre-adolescent and adolescent students so that we can increasingly deepen our knowledge about the development of reading skills in students in the final years of basic education.

## Data Availability

The raw data supporting the conclusions of this article will be made available by the authors, without undue reservation.
